# Developing Paper Based Diagnostic Technique to Detect Uric Acid in Urine

**DOI:** 10.3389/fchem.2018.00496

**Published:** 2018-10-17

**Authors:** Md. Nazibul Islam, Isteaque Ahmed, Muzahidul Islam Anik, Md. Sakib Ferdous, Mohidus Samad Khan

**Affiliations:** Department of Chemical Engineering, Bangladesh University of Engineering and Technology, Dhaka, Bangladesh

**Keywords:** uric acid, renal dysfunction, paper diagnostics, colorimetric detection, reaction kinetics

## Abstract

Urinary or serum uric acid concentration is an indicator of chronic kidney condition. An increase in uric acid concentration may indicate renal dysfunction. Reliable instantaneous detection of uric acid without requiring sophisticated laboratory and analytical instrumentation, such as: chromatographic and spectrophotometric methods, would be invaluable for patients with renal complication. This paper reports the early development of a simple, low-cost, instantaneous and user-friendly paper based diagnostic device (PAD) for the qualitative and quantitative detection of uric acid in urine. A colorimetric detection technique was developed based on the intensity of Prussian blue color formation on paper in presence of uric acid; the reaction rate of corresponding chemical reactions on paper surface was also studied. Based on the colorimetric signal produced on paper surface, a calibration curve was developed to detect unknown concentration of uric acid in urine. The effect of temperature on formation of color signal on paper surface was also analyzed. In this study, estimation of urinary uric acid using MATLAB coding on a windows platform was demonstrated as the use of software application and digital diagnostics. This paper-based technique is faster and less expensive compared to traditional detection techniques. The paper-based diagnostic can be integrated with a camera of smart phone, tablet computer or laptop and an image processing application (using windows/android/IOS platform) as a part of digital diagnostics. Therefore, with proper calibration, the paper-based technique can be compatible and economical to the sophisticated detection techniques used to detect urinary uric acid.

## Introduction

Uric acid is the final byproduct of purine metabolism for human (Heinig and Johnson, 2006; Amir et al., 2011). A change in dietary behavior, such as: an increase in consumption of purine rich foods e.g., meat and seafood, sugar-sweetened beverages, and alcohol, can result in an increase in uric acid levels in urine and blood (Heinig and Johnson, 2006; Lowl et al., 2010; Roddy and Dohert, 2010; Amir et al., 2011; Rho et al., 2011). In addition, an increase in uric acid may indicate defects of uric acid transport in the nephron and renal under-excretion of uric acid (Perez-Ruiz et al., 2002; Heinig and Johnson, 2006). A higher level of serum uric acid (>7 mg/dl for men and >6.5 mg/dl for women) or urinary uric acid (>700 mg/day) is known as hyperuricemia (Perez-Ruiz et al., 2002; Heinig and Johnson, 2006; Siu et al., 2006; Chonchol et al., 2007; Mohandas and Johnson, 2008; Lowl et al., 2010; Amir et al., 2011). Hyperuricemia may disrupt renal function by causing renal arteriolar changes and glomerular damage which may lead to *de novo* renal disease as well as accelerate existing renal diseases (Perez-Ruiz et al., 2002; Heinig and Johnson, 2006; Siu et al., 2006; Chonchol et al., 2007; Mohandas and Johnson, 2008). Prolonged occurrence (> 3 months) of renal dysfunction can lead to chronic kidney disease (CKD) (Siu et al., 2006; Chonchol et al., 2007; Jha et al., 2013). In 2010, CKD was ranked 18th in the list of the “causes of total number of global deaths,” which was ranked 29th back in 1990 (Jha et al., 2013). According to the World Health Organization (WHO), in 2012, an estimated 864,226 deaths (1.5% of deaths worldwide) were attributed to CKD (Webster et al., 2016). In addition, hyperuricemia is a precursor of gout, and is associated with the development of type 2 diabetes, obesity, Lesch-Nyhan syndrome, and arteriolar hypertension which may lead to cardiovascular disease (Perez-Ruiz et al., 2002; Bos et al., 2006; Heinig and Johnson, 2006; Siu et al., 2006; Chonchol et al., 2007; Dehghan et al., 2008; Mohandas and Johnson, 2008; Lowl et al., 2010; Amir et al., 2011). However, early detection of kidney disease can reduce the risk of kidney failure progression and cardiovascular disease by up to 50% (Biljak et al., 2015). Since uric acid concentration can be used as a biomarker to monitor renal health (Perez-Ruiz et al., 2002; Chonchol et al., 2007; Mohandas and Johnson, 2008; Lowl et al., 2010), a rapid, reliable, and low cost technique to detect uric acid from human biofluids will be beneficial to patients and physicians.

Existing analytical methods to detect uric acid in biofluids include spectroscopy, chromatography, electrochemistry, membrane and capillary electrophoresis (Gochman and Schmitz, 1971; Kageyama, 1971; Kabasakalian et al., 1973; Inoue et al., 2003; Perelló et al., 2005; Cooper et al., 2006; Arora et al., 2009; Uricase/PAP test, 2009; Bhawna and Pundir, 2010; Rocha and Rocha, 2010; Amir et al., 2011; Sanchez et al., 2011; Sidorova and Grigoriev, 2012; Hamzah et al., 2013; WitkowskaNery et al., 2016). Most conventional spectrophotometric methods for uric acid detection involve colorimetric enzymatic reaction, where. uric acid is oxidized in the presence of uricase and forms allantoin (C4H6N4O3), carbon dioxide and hydrogen peroxide (Gochman and Schmitz, 1971; Kageyama, 1971; Kabasakalian et al., 1973; Uricase/PAP test, 2009; Rocha and Rocha, 2010; Hamzah et al., 2013). In addition, researchers have reported a spectrophotometric method based on the reduction of Cu(II) ions to Cu(I) ions in presence of urinary uric acid (Rocha and Rocha, 2010; Sanchez et al., 2011). Amir et al. have reported a flow injection spectrophotometric method based on the reduction of potassium ferricyanide in presence of uric acid to potassium ferrocyanide (Amir et al., 2011). Arora et al. have developed an uricase bound membrane coupled with dissolve oxygen (DO) metric uric acid biosensor (Arora et al., 2009; Bhawna and Pundir, 2010). WitkowskaNery et al. have demonstrated a electrochemical biosensor integrated with platinum electrode, enzyme immobilized bioactive paper channel, buffer filled sponge and pencil drawn pseudo-reference electrode to detect glucose and uric acid (WitkowskaNery et al., 2016). There are other studies, which have reported uric acid detection from biofluids (saliva, serum and urine) using high-performance liquid chromatography (HPLC) technique (Jen et al., 2002; Inoue et al., 2003; Perelló et al., 2005; Cooper et al., 2006). The principles, techniques, types of biofluid samples and detection range of different methods of uric acid detection are enlisted in Table 1. These techniques often require sophisticated equipment and out sourcing of samples to specialized laboratories, which makes these detection techniques expensive and time consuming. Hence, there is a need for instantaneous and low-cost point-of-care detection technique capable for qualitative and quantitative detection of uric acid in human biofluids. Paper based diagnostics, often used for health and environmental purposes, can offer an attractive option for detecting biomarkers such as uric acid from biofluids such as urine, serum, etc. (Khan et al., 2009, 2010c, 2015, 2016, 2018a; Shen et al., 2009; Koh et al., 2016; Zhang et al., 2017).

**Table 1:** Different detection methods of uric acid from biofluids (Gochman and Schmitz, 1971; Kageyama, 1971; Kabasakalian et al., 1973; Inoue et al., 2003; Perelló et al., 2005; Cooper et al., 2006; Arora et al., 2009; Uricase/PAP test, 2009; Bhawna and Pundir, 2010; Rocha and Rocha, 2010; Amir et al., 2011; Sanchez et al., 2011; Sidorova and Grigoriev, 2012; Hamzah et al., 2013; WitkowskaNery et al., 2016).

**Principle**	**Technique**	**Biofluid sample**	**Detection range (ppm)**	**References**
Enzymatic Uricase Method with 4-Aminodiphenylamine Diazonium Sulfate	Spectrophotometric	Urine	84–2185	Hamzah et al., 2013
Enzymatic Uricase Method with 4-Aminoantipyrine 1 mmol/lPeroxidase	Spectrophotometric	Urine, Serum	0–303	Uricase/PAP test, 2009
Enzymatic Uricase-Catalase Method	Spectrophotometric	Urine, Serum	0–319	Kageyama, 1971
Enzymatic Uricase-Peroxidase Method coupled with 3-methyl-2-benzothiazolinone hydrazone and N,N-dimethylaniline	Spectrophotometric	Serum	0–160	Gochman and Schmitz, 1971
Enzymatic Uricase Method with Tribromophenol-Aminoantipyrine Chromogen	Spectrophotometric	Serum	0–319	Kabasakalian et al., 1973
Reduction of Potassium ferricyanide and formation of Prussian blue	Spectrophotometric	Urine, Serum	0–185	Rocha and Rocha, 2010; Sanchez et al., 2011
Reduction of Cu(II) ion and complexation with 4,4′-Dicarboxy-2,2′-Bichinoline (BCA)	Spectrophotometric	Urine	2–17	Amir et al., 2011
Biosensor based on Uricase bound PVC membrane	Enzyme-membrane biosensor	Serum	0–100	Arora et al., 2009; Bhawna and Pundir, 2010
capillary zone electrophoresis	Capillary Electrophoresis	Urine	42–168	Sidorova and Grigoriev, 2012
Electrochemical detection	Electrochemical	Proof of concept	17-200	WitkowskaNery et al., 2016
high-performance liquid chromatography	Chromatographic	Saliva, Serum, Urine	1–27	Jen et al., 2002; Inoue et al., 2003; Perelló et al., 2005; Cooper et al., 2006

This article reports the early development of a simple, low-cost and user-friendly paper-based diagnostic device (PAD) to detect uric acid in urine. For the proposed device, ferric chloride and potassium ferricyanide solutions were used as reagents. A color chemistry based on the reaction between ferric chloride, uric acid and potassium ferricyanide was used to develop the paper based diagnostic device. In presence of uric acid in biofluid such as urine, Prussian blue color is formed on the paper surface (Adhikamsetty and Jonnalagadda, 2009). Reaction rate of this reaction mechanism on paper surface was also studied. The color formed on paper surface with respect to uric acid concentration was analyzed using image analysis technique^1^. A calibration curve was developed based on the intensity of color formation on paper surface corresponding to known uric acid concentrations. The effect of temperature on color formation on paper was also analyzed. A MATLAB code was generated to demonstrate uric acid detection using computer/cell phone based applications (windows, android or IOS platform). The proposed technique will provide patients living in remote areas and patients with chronic kidney diseases the ability to assess and report urinary uric acid concentration at low cost and on regular basis. This technique will also facilitate physicians to monitor, diagnose and medicate kidney diseases (Khan et al., 2018b).

## Reaction mechanism and kinetics

Ferric chloride reacts with potassium ferricyanide in presence of uric acid to form Prussian blue. Paper surface was treated with potassium ferricyanide followed by uric acid sample. Ferric chloride solution was introduced to this treated paper to complete the reaction. The reaction scheme is shown below (Silverman and Gubernick, 1946; Izatt et al., 1970; Hill, 1977; Peters, 2008; Adhikamsetty and Jonnalagadda, 2009):

At first, potassium ferricyanide reacts with uric acid (C_5_H_4_N_4_O_3_) to form potassium ferrocyanide (Equation 1; Silverman and Gubernick, 1946).

(1) $$\underset{\begin{array}{l} \text{Uricacid} \end{array}}{{\text{C}}_{5}{\text{H}}_{4}{\text{N}}_{4}{\text{O}}_{3}}+\underset{\begin{array}{l} \text{Potassium}\\ \text{ferricyanid} \end{array}}{{\text{K}}_{3}[\text{Fe}{\left(\text{CN}\right)}_{6}]}➝\underset{\begin{array}{l} \text{Potassium}\\ \text{ferrocyanide} \end{array}}{{\text{K}}_{4}[\text{Fe}{\left(\text{CN}\right)}_{6}]}$$

Potassium ferrocyanide then reacts with ferric chloride to form Prussian blue (Izatt et al., 1970; Peters, 2008). This reaction takes place in several steps; potassium ferrocyanide reacts with Fe^3+^ ion to form HFe[Fe(CN)_6_]. There are major and minor paths for HFe[Fe(CN)_6_] formation (Izatt et al., 1970; Peters, 2008).

Major paths:

(2) $$\begin{array}{c} \text{F}{\text{e}}^{3+}+{\left[\text{Fe}{\left(\text{CN}\right)}_{6}\right]}^{4-}\overset{{\text{k}}_{1}}{\underset{{}_{{\text{k}}_{-1}}}{⇄}}\text{Fe}{\left[\text{Fe}{\left(\text{CN}\right)}_{6}\right]}^{-}(\text{Fast}) \end{array}$$

$$\begin{array}{c} {\text{H}}^{+}+\text{Fe}{\left[\text{Fe}{\left(\text{CN}\right)}_{6}\right]}^{-}\overset{{\text{k}}_{2}}{➝}\text{HFe}[\text{Fe}{\left(\text{CN}\right)}_{6}] \end{array}$$

(3) $$\begin{array}{r} \;\left(\text{Slow}\right)\text{ Rate limiting} \end{array}$$

Minor Paths:

(4) $$\begin{array}{c} {\text{H}}^{+}+{\left[\text{Fe}{\left(\text{CN}\right)}_{6}\right]}^{4}\overset{{\text{k}}_{3}}{\underset{{\text{k}}_{-3}}{⇄}}\text{H}{\left[\text{Fe}{\left(\text{CN}\right)}_{6}\right]}^{3-} \end{array}$$

(5) $${\text{Fe}}^{\text{3+}}\text{+H}{\left[\text{Fe}{\left(\text{CN}\right)}_{\text{6}}\right]}^{\text{3-}}\overset{{\text{k}}_{\text{4}}}{➝}\text{HFe}[\text{Fe}{\left(\text{CN}\right)}_{\text{6}}](\text{Slow})$$

After the formation of HFe[Fe(CN)_6_], three molecules of HFe[Fe(CN)_6_] and one Fe^3+^ion assemble to form insoluble Prussian blue (Izatt et al., 1970).

(6) $$3\text{HFe}[\text{Fe}{\left(\text{CN}\right)}_{6}]+\text{F}{\text{e}}^{3+}\overset{{\text{k}}_{5}}{\underset{{}_{{\text{k}}_{-5}}}{⇄}}\underset{\begin{array}{c} \text{Prussian}\\ \text{blue} \end{array}}{\text{F}{\text{e}}_{4}{\left[\text{Fe}{\left(\text{CN}\right)}_{6}\right]}_{3}}+3{\text{H}}^{+}$$

Assuming Quasi-equilibrium condition, Equations 2, 4 can be considered very fast reactions, which have high reaction rates, reach equilibrium rapidly and this equilibrium is not affected by neighboring reactions (Hill, 1977). Equation 3 can be regarded as the slowest step which governs the entire process (rate-limiting step).

Thus, the reaction kinetics can be expressed as:

(7) $$\begin{array}{c} {\text{H}}^{+}+\text{Fe}{\left[\text{Fe}{\left(\text{CN}\right)}_{6}\right]}^{-}➝\text{HFe}[\text{Fe}{\left(\text{CN}\right)}_{6}] \end{array}$$

*t* = 0    a        0

*t* = t    a-x    x

From the graphical representation of the following equations, the reaction order of the above mechanism can be assessed (Bahl et al., 2009):

Zero order:       x = *K*t

First order        2.303×log (a-x) = - *K*t + c

Second order    1/(a-x) = *K*t + c

## Methodology

### Materials

Reagent grade uric acid (Sigma-Aldrich, United States)^2^, potassium ferricyanide (Carl Roth GmbH, Germany)^3^, ferric chloride (Loba-chemie, India)^4^, ultra-high purity de-ionized water (18.2MΩ.cm, Purite, United Kingdom), and whatman#1 filter paper were used in this study. The artificial urine was prepared in the laboratory (Martinez et al., 2007, 2009). Images used to analyze reaction kinetics were captured using a Sony Alpha 57 DSLR camera (Sony Corp., Japan). The temperature and humidity were measured using Palmer Hygrometer (Palmer, USA). For calibration curve, the color produced on the paper surface at 25°C (room temperature) was measured at 600 dpi using a standard scanner (canon lide 120). The images were analyzed using ImageJ software (ImageJ 1.47t). ImageJ calculates the weighted average gray value within a selected section of an image which can be related to the concentration of uric acid on the activated paper (Khan and Garnier, 2014; Khan et al., 2015). A phoenix RSM 65 H hot plate (Phoenix instruments, Germany) was used to measure relationship between changes in color intensity with respect to temperature.

### Method

**Figure 1:**
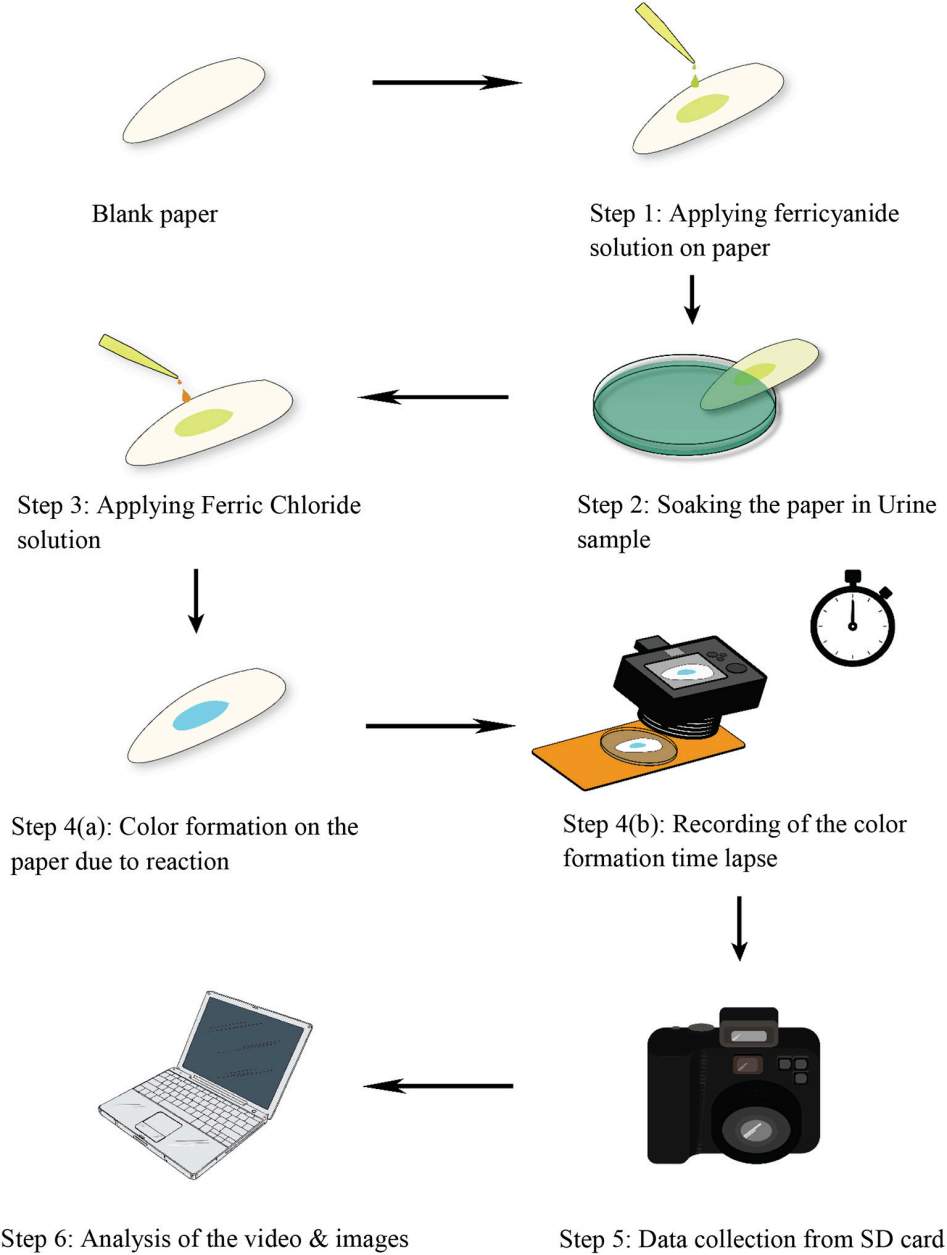
Schematic representation of steps involved to develop paper-based technique to detect uric acid and to study reaction kinetics of corresponding chemical reactions on paper surface.

Using a micropipette, 30 μL potassium ferricyanide solution (0.01 M) was applied on Whatman#1 filter paper (Diameter: 2 cm). Then, 2X diluted urine sample was applied on treated paper to make the paper sample saturated. Uric acid in urine reacted with potassium ferricyanide to form potassium ferrocyanide. Finally, 30 μL ferric chloride solution (0.01 M) was applied on the treated paper. Prussian blue color was formed on paper surface because of reaction between ferric chloride and potassium ferricyanide (Figure 1). Figure 1 represents schematic diagrams of treating paper surface with different reagents to develop paper diagnostics for uric acid detection. The sensitivity of the test can be improved by increasing the dilution factor of urine sample (2X−5X).

#### Study of reaction order and kinetics

To determine the reaction order and kinetics, images were captured using a Sony Alpha 57 DSLR camera on video mode. The video clips were transferred to a computer and converted into JPEG files (30 frames per second) using a video to JPEG converter (v. 5.0.99, DVDVideoSoft Ltd. United States). The converted images were analyzed using ImageJ software (ImageJ 1.41t). ImageJ calculates the gray values of RGB (red-green-blue) images. RGB pixels are converted to gray values using the built-in formula (gray = (red + green + blue)/3) (Khan and Garnier, 2014; Khan et al., 2015). The RGB color scheme uses a numbering scale ranging from 0 to 255 where the “black” is numbered 0 and the “white” is numbered 255, which means the lower the number, the darker the color. On the other hand, in the CMY (cyan-magenta-yellow) color scheme the “black” is numbered 255 and the “white” is numbered 0, which means the higher the number the darker the color (Khan and Garnier, 2014; Khan et al., 2015). In the formation of Prussian blue, high concentration of uric acid yields darker color i.e. the darker the color, the higher the uric acid concentration. To correlate the intensity of this color formation with color scheme numbering, the RGB gray values were converted into CMY gray values: CMY = 255-RGB (Khan and Garnier, 2014; Khan et al., 2015). Therefore, the gray value results presented in this article are CMY gray values i.e., the darker the color, the higher the gray values and, the higher the concentration of uric acid.

#### Qualitative and quantitative detection of uric acid

To develop a calibration curve for the qualitative and quantitative determination of uric acid in urine, artificial urine (Martinez et al., 2007, 2009) containing different concentrations of uric acid was applied on potassium ferricyanide treated paper samples. Then, ferric chloride was applied on the treated paper samples to form color signals on paper surface. These color signals were scanned and analyzed using standard image processing software (ImageJ 1.41t). The intensity of the color signals produced from the reaction of uric acid and activated paper samples varied according to the concentration of the uric acid in urine solution. Finally, a calibration curve was developed for the qualitative and quantitative detection of uric acid in urine.

#### Analyzing the effect of temperature

A hot plate (phoenix RSM 65 H hot plate) was used to analyze the effect of temperature on color signal. The temperature of the plate can be set at a desired level. At different set temperatures (25, 30, 35, 40, 45 and 50°C), potassium ferricyanide treated paper samples (Whatman#1, Diameter: 2 cm) were placed on the plate. To attain equilibrium, the paper samples were kept on the plate for 10 min. Subsequently, steps described in Figure 1 were performed on the paper and the resultant color signal was analyzed.

### Computer/cell phone based application in health monitoring

To demonstrate the concept of windows, android or IOS based application in health monitoring, a MATLAB (Mathworks, r2013a) code was developed for digital analysis of proposed paper based diagnostic kit. Image analysis tool of MATLAB can analyze the color intensity of any image and produce a three-dimensional matrix, which quantifies the color intensity at different points (depending on the resolution of the image, 600 DPI in this case) of the image in RGB (Red, green and blue). A code was generated to calculate uric acid concentration from this matrix. This code can be used as a baseline to develop any windows, android or IOS based application integrated with paper diagnostics to detect and report uric acid concentration in urine or any other biofluid.

## Results and discussion

### Qualitative and quantitative detection of uric acid in urine sample

**Figure 2:**
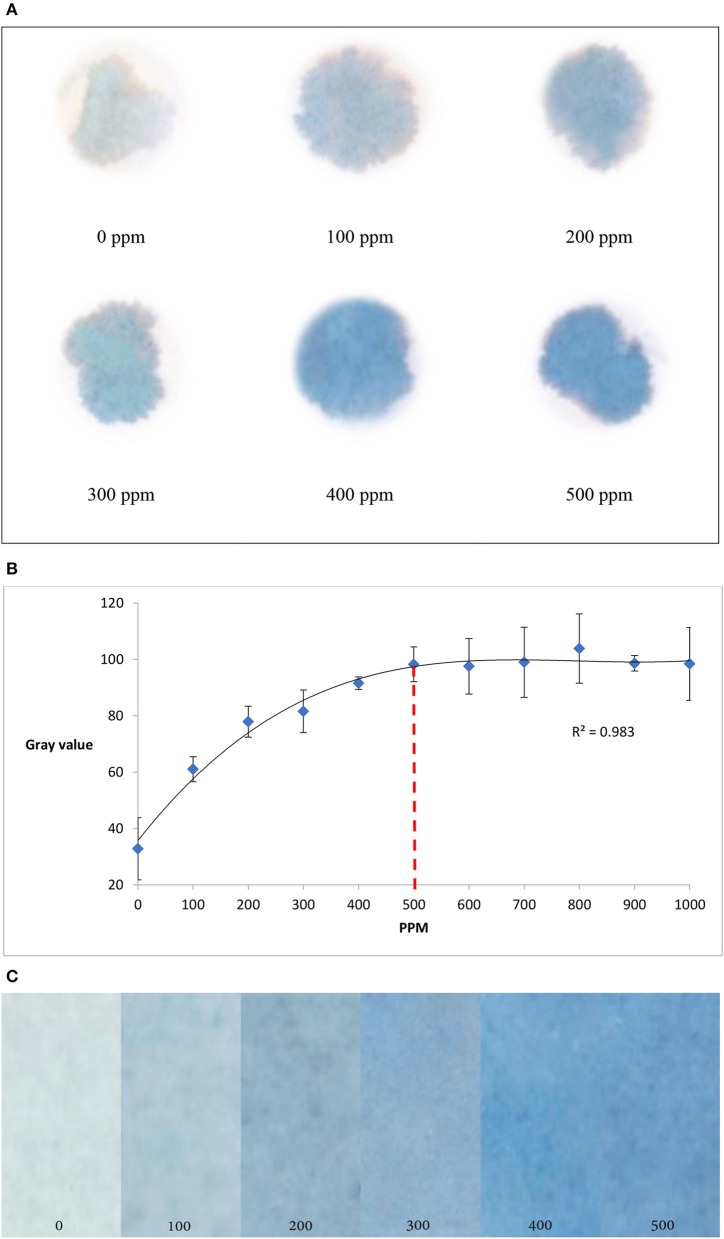
Detection of uric acid concentration in urine using paper diagnostics. **(A)** Experimental detection of different uric acid concentrations. **(B)** Change in color intensity (measured in gray value) with respect to the change in uric acid concentration. From the graph it is seen that the color intensity reaches saturation at around 500 ppm of uric acid concentration. (Standard deviation bars for *n* = 4 samples) **(C)**. The color gradient shows that higher the concentration, the darker is the color.

To develop paper based diagnostic device (PAD) to detect uric acid in urine, paper samples were treated with potassium ferricyanide (0.01 M, 30 μL), and urine sample was applied on the paper surface. After that, ferric chloride (0.01 M, 30 μL) was added onto the paper samples. Uric acid in urine sample reacted with ferricyanide solution to produce ferrocyanide which then reacted with ferric chloride and produced Prussian blue color signals on the paper surface (Figure 1). The higher the uric acid concentration in urine sample, the stronger the color signals it produced on paper. Figure 2A demonstrates experimental results of qualitative detection of uric acid in urine sample using paper diagnostics.

Color signals formed for different concentrations of uric acid in urine solutions (Figure 2A) were scanned and analyzed using ImageJ software (approximately 100 mm^2^ at 12.2 pixels/mm)^1^. To understand the change in color intensity with respect to uric acid concentration, corresponding CMY (cyan, magenta and yellow) values of the color signals were plotted against uric acid concentrations (Figure 2B). From Figure 2B, it is seen that the color intensity reaches saturation at around 500 ppm of uric acid concentration. The experimental results produce a color gradient indicating uric acid concentration (Figure 2C): the higher the concentration, the darker the color product formation.

### Reaction rate

**Figure 3:**
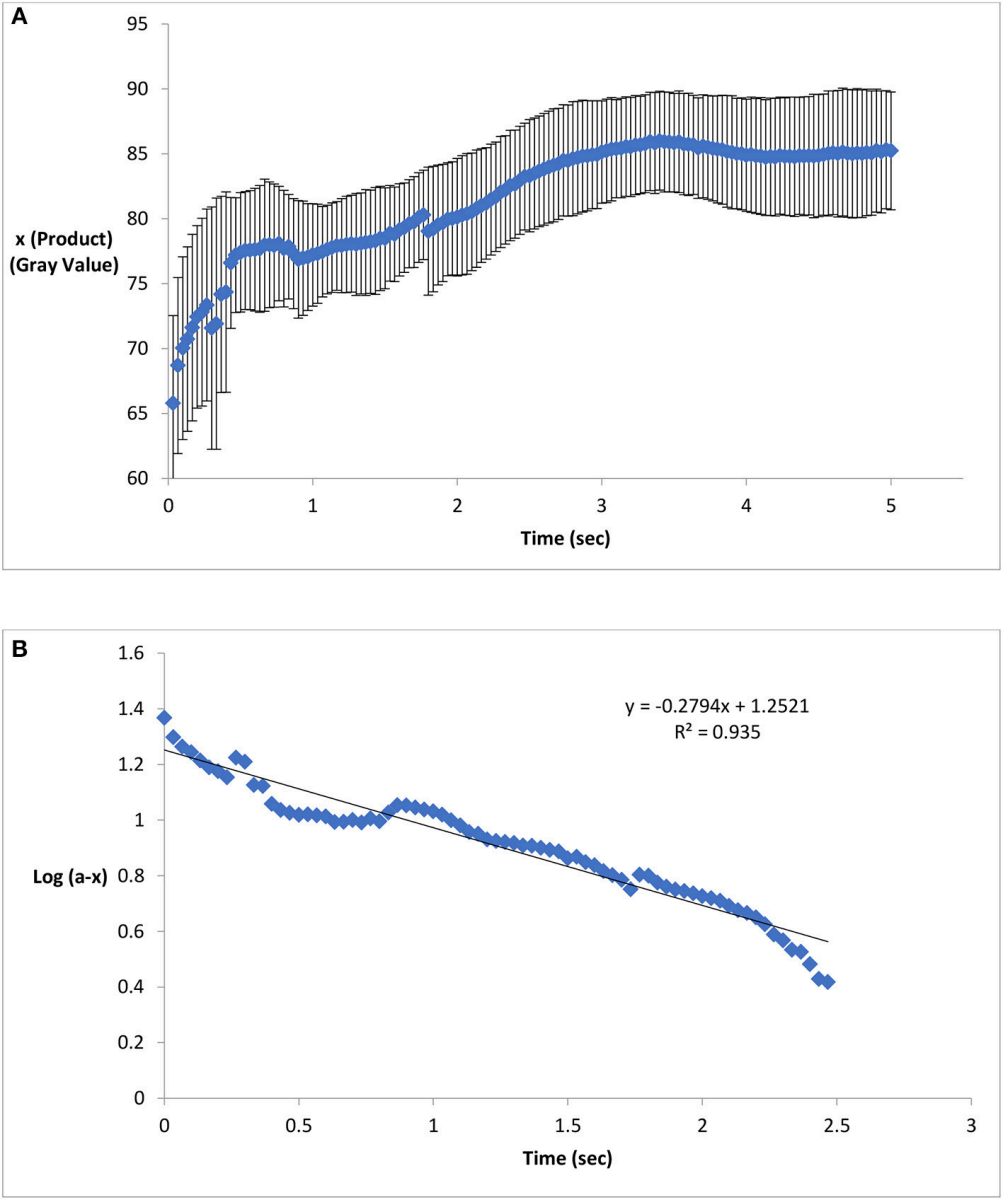
Product formation and reaction kinetics on chemical treated paper surface. **(A)** Formation of Prussian blue on activated paper as a function of time. The color intensity of the product increased non-linearly as a function of time. **(B)** A straight line of Log (a-x) vs. time graph conforms first order reaction kinetics and the slope of this straight line indicates the reaction rate (Standard deviation bars for *n* = 5 samples).

Figure 3 illustrates the reaction kinetics of color formation on paper surface. Figures 3A indicates the formation of Prussian blue color (x) on activated paper as a function of time. The color intensity of the product increased non-linearly as a function of time. Figure 3B graphically represents log (a-x) as a function of time; the straight line of log (a-x) vs. time graph conforms that the reaction follows first order reaction mechanism. From Figure 3B, the reaction rate constant (*K*), was calculated as 0.64 s^−1^. The calculated value is of the same order of magnitude with reported result (0.4–0.47 s^−1^; 25–30°C) based on reaction in liquid solution (Adhikamsetty and Jonnalagadda, 2009).

### Effect of temperature

**Figure 4:**
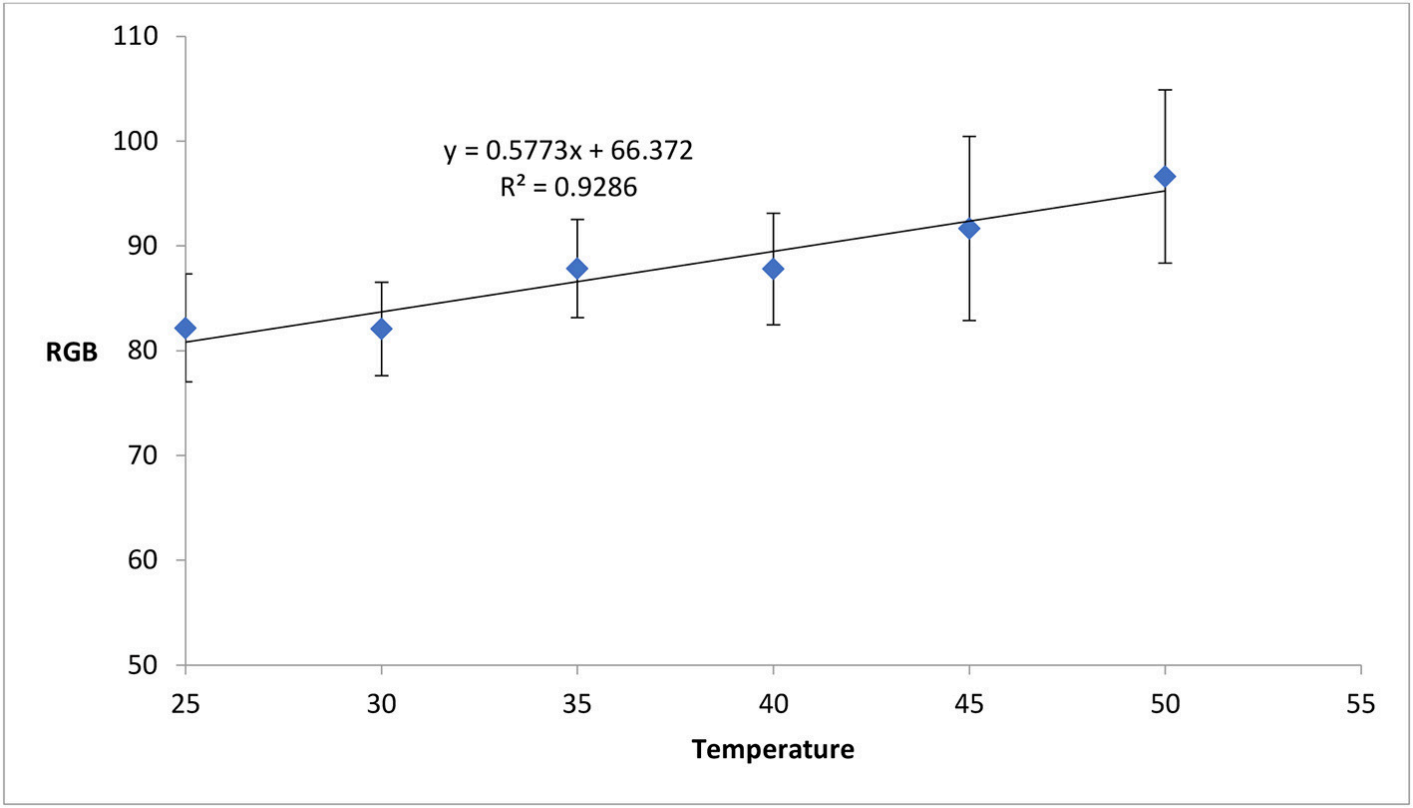
Change in color intensity (measured in gray value) with respect to temperature. The color intensity increases with the increase in temperature. (Standard deviation bars for *n* = 4 samples). Paper yellowing effect at high temperature can be probable cause for this increase in color intensity with temperature.

To determine the effect of temperature on color formation on paper, potassium ferricyanide treated paper samples were kept at different temperatures and concentration of uric acid sample was tested using those treated papers at corresponding temperatures. The color intensity was found to increase with respect to the increase in temperature (Figure 4). An increase in temperature from 25° to 50°C changes the RGB value from 82 to 96. A probable reason of the increase in color intensity on paper surface could be the paper yellowing effect at high temperature (Bigourdan and Reilly, 2002; Khan et al., 2010a,b; Karlovits and Gregor-Svetec, 2012). Correction factor of temperature or temperature specific calibration curve can be used to render accurate results.

### Laboratory trial

The paper diagnostic technique was used to detect unknown uric acid concentration in urine. Twenty-four-hour urine samples were collected from three male subjects aged between 20 and 25 years. The uric acid concentrations of these urine samples were then measured using the proposed paper-based technique. The calibration curve (Figure 2B, 0–500 ppm) was used to quantify uric acid concentrations for corresponding color signals. Pathological laboratory trials were also performed to determine serum uric acid using existing diagnostic technique (Modified Fossati method; Fossati et al., 1980). In this method, H_2_O_2_ produced in enzymatic oxidation of uric acid, reacts with 4-aminoantipyrine (4-AAP) in the presence of N,N-bis(4-sulfobutyl)-3,5-dimethylaniline, disodium salt (MADB) to produce a blue dye which is then measured using a spectrophotometer (Fossati et al., 1980; Coulter, 2009). The serum uric acid concentrations were then converted into urinary uric acid concentration (Sila-On et al., 1991). Pathological laboratory trial data were compared to corresponding PAD trial data (Table 2). The results were also compared with published data (Putnam, 1971; Lowl et al., 2010; Amir et al., 2011). Table 2 shows that pathological laboratory trial data and PAD trial data are compatible and within acceptable limit (Putnam, 1971; Lowl et al., 2010; Amir et al., 2011); PAD data for patient 3 was higher than that of pathological laboratory data; however, both are within acceptable limit (300–700 ppm).

**Table 2:** Validity test of the paper based uric acid detection technique.

**No**.	**Paper based technique (ppm) (*n* = 3)**	**Pathological trial (ppm)**	**Published data (ppm) (Putnam, 1971; Lowl et al., 2010; Amir et al., 2011)**
1	550 ± 56	553	300–700
2	558 ± 17	595	
3	442 ± 27	315	

## Application based detection

**Figure 5:**
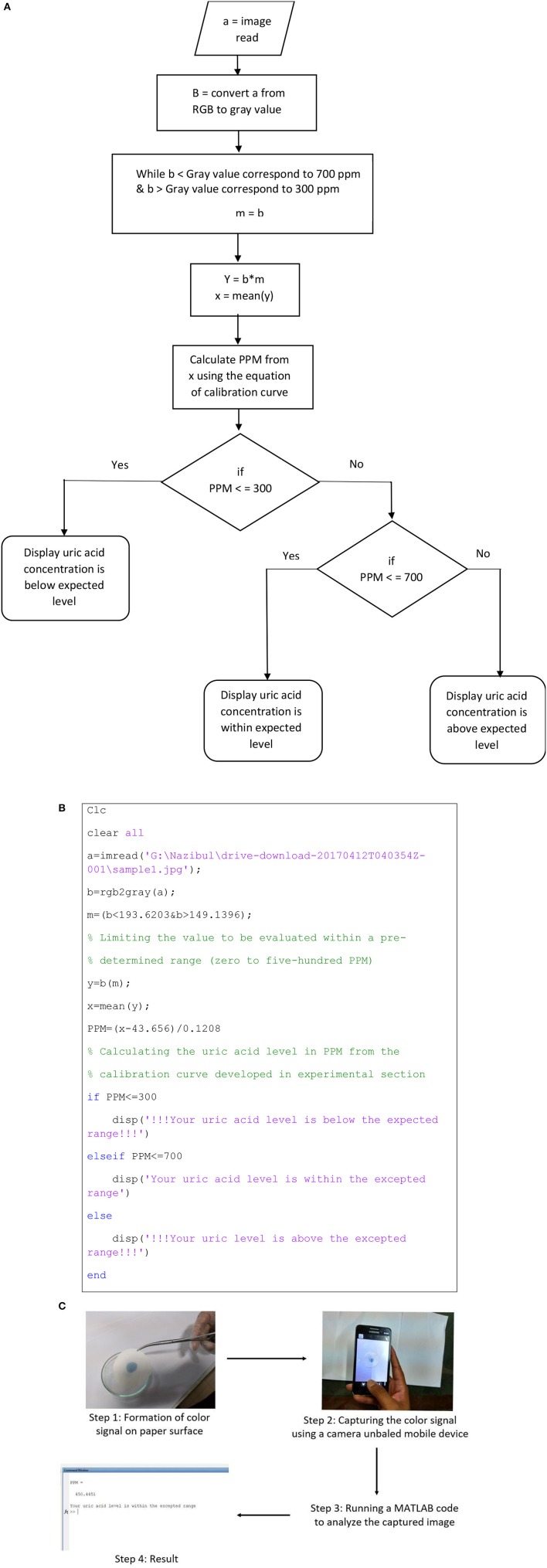
Demonstration of application-based detection. **(A)** Algorithm for the MATLAB code. **(B)** MATLAB code for color signal analysis. **(C)** Estimation of urinary uric acid using a mobile device.

The proposed diagnostic technique can be integrated with windows, android or IOS based applications to detect and report uric acid concentration in urine. The color signal produced on the paper, can be analyzed with the help of a camera enabled mobile devices, computers or electronic gadgets. The results can be stored in the device to monitor urinary uric acid trend with time and can be sent to an established medical facility for further analysis. Figure 5A describes the algorithm for the code; Figure 5B illustrates a MATLAB code which can be used as a baseline to develop any mobile application. According to the algorithm, the image is at first inserted into the MATLAB program. MATLAB reads the image in RGB (Red, Green and Blue) format, which is displayed as a 3-dimensional matrix. This 3D matrix is then converted into a 2D matrix by converting the RGB image to Gray image (Black and White image). Outliers from this matrix are then sorted and eliminated. The mean value from the resulting matrix is taken for further calculation. Equation developed from the calibration curve is used to convert the color value (mean value) into concentration value as parts per million (ppm) of uric acid. Finally, this value is displayed with a concluding remark. Figure 5C shows the estimation of urinary uric acid using a mobile device.

## Conclusion

This article presents the development of the first-generation point-of-care paper-based detection technique for qualitative and quantitative detection of uric acid in urine. The reaction kinetics of corresponding chemical reactions and effect of temperature on color formation mechanism on paper surface was also analyzed. It was found that the mechanism follows first order reaction kinetics and temperature may affect the colorimetric technique. A calibration curve was developed to measure the uric acid present in urine from color intensity. As a proof of concept, the uric acid content in the urine of adult males was measured using proposed PAD, which were compatible with laboratory trial data and within acceptable limit reported in literature. This technique can also be integrated as a part of digital diagnostics with the help of a camera of smart phone, tablet computer or laptop, and image processing software. Estimation of urinary uric acid using MATLAB coding on a windows platform was demonstrated as the use of software application and digital diagnostics.

The proposed paper diagnostic for uric acid detection in urine and other biofluids is cheap, biocompatible, biodegradable, and can give results in few seconds. Using the proposed technique, patients can regularly (daily or weekly) monitor urinary uric acid level at home. If the urinary uric acid level is frequently found close to or higher than the acceptable limit, the patients can take necessary steps to reduce uric acid level by changing their diets and lifestyle, or by consulting a doctor for further analysis and advice. This technique will also facilitate physicians and dietitians to monitor and medicate kidney conditions of their patients.

## Ethics statement

Research and manuscript are original and unpublished. All authors read and approved the final manuscript. The authors followed the instructions from university medical center and local pathological laboratories while collecting and testing urine samples from volunteers. The volunteers gave signed consent letters as a sign of agreement to provide urine samples to support and validate the study.

## Author contributions

MI carried out a major part of the literature review, experimental studies and drafted the manuscript. IA, MA, and MF carried out literature review and performed laboratory experiments. IA also helped developing MATLAB code. MK conceived the study, supervised the research project and manuscript preparation, and helped to revise and finalize the manuscript. The final manuscript was read and approved by all the authors.

### Conflict of interest statement

The authors declare that the research was conducted in the absence of any commercial or financial relationships that could be construed as a potential conflict of interest.

## Footnotes

^1^*Image Processing Toolbox*, ™, *User's Guide*. 2017, The MathWorks, Inc.

^2^*Uric acid product specification*. Sigma-Aldrich. Editor.

^3^*Potassium hexacyanoferrate (III)*, Specification, C.R. GmbH, Editor.

^4^*Ferric chloride (hexahydrate) AR/ACS MSDS CAS: 010025-77-1*. (2012). Loba Chemie.
